# Continuous Non-Invasive Cardiac Output: Myth or Reality

**DOI:** 10.5935/abc.20190163

**Published:** 2019-08

**Authors:** João Manoel Rossi Neto

**Affiliations:** Instituto Dante Pazzanese de Cardiologia, São Paulo, SP - Brazil; Grupo Fleury, São Paulo, SP - Brazil

**Keywords:** Cardiac Ouput, Electric, Impedance, Pharmacological, Interventions, Breathing Exercise

Cardiac output (CO) is an important cardiovascular system function parameter. Changes in cardiac function are commonly observed as a response to physical training and pharmacological interventions.^1^ Unfortunately, the methods for assessing CO are invasive, leading to well-known complications and considered inconvenient in daily practice.^2^ For this reason, the search for new noninvasive methods that can accurately detect CO at rest, at physical exertion or as a response to a clinical intervention has become desirable in academic and non-academic circles. The ideal method for measuring CO at rest and during exercise should be noninvasive, safe, reproducible and inexpensive.^3^

The Cardiopulmonary Exercise Testing (CPT) is recommended in the evaluation of cardiorespiratory fitness and exercise tolerance in athletes, the general population and in patients.^4^ Briefly, CO and systolic volume can be estimated during CPT through measured VO_2_.^5^ In 2001, Williams et al.^6^ were the first ones to integrate CPT with non-invasive measures of CO using rebreathing (RB) of carbon dioxide, but the technique was quickly abandoned due to its difficulty and inaccuracy. Another non-invasive method is thoracic electrical bioimpedance (TEB), first described in 1966 by Kubicek et al.,^7^ which measures thoracic resistance as a result of changes in blood velocity during the cardiac cycle and uses an algorithm to calculate the CO.

Another promising technique is based on thoracic bioreactance (TB) (NICOM^TM^, Cheetah Medical Inc., Wilmington, DE), which analyzes the variations in beat-to-beat tension after a high-frequency transthoracic current is applied. This device records the electric current phase in the thorax. The systolic volume is directly proportional to the phase displacement.^8^ Despite some controversial studies, this technique seemed to be more reliable.^8-10^ It is worth mentioning that the CO measurement is simple to perform and does not require patient cooperation, both at rest and at the exercise peak. It should be noted that some conditions, such as significant pleural effusion, have a negative impact on the accuracy of this method.^11^

In a meta-analysis, the percentage errors for CO monitoring devices were 42% for TEB and TB, 40% for RB of carbon dioxide and 62% for the methods of pulse wave analysis.^12^ The most recent meta-analysis that evaluated adult and pediatric patients in different clinical situations (mostly in the hospital setting), it was demonstrated that TEB accuracy showed high heterogeneity between the studies and that the mean percentage error grouped in all the subgroups was above the acceptable 30%. Therefore, TEB could not replace thermodilution and transthoracic echocardiography for the measurement of absolute CO values.^13^

Okwose et al.^14^ showed that the RB of an inert gas and TB methods had acceptable levels of agreement to estimate the CO at higher degrees of metabolic demand during a CPT. However, they concluded they could not be used interchangeably because of the great disparity in results at rest and in low-to-moderate intensity exercises. Unlike this study, Torto et al.^15^ showed that cardio-impedance could be less ideal for supramaximal exercise intensities.

In this issue, Coll et al.^16^ evaluated the test-retest reliability of CO and cardiac work during CPT by TB in healthy adults under routine clinical conditions in an uncontrolled environment.

They concluded that, according to the findings, there is an obstacle to the clinical use of TB in healthy individuals whereas outliers are not identified (32% of the initial sample). That is, under routine clinical conditions, almost one-third of the patients showed measurement errors and, according to the authors, these outliers were probably due to an underlying technical reason; thus, further improvements in TB are required, such as regarding the use and the quality of the electrodes. This study contested the results of the study by Jones et al.,^11^ which had demonstrated that TB could be viable under strict control conditions and in the research environment.

The results of the studies published to date showed that even in situations of in-hospital use and controlled environment (anesthesia, intensive care and even outpatient clinics) in which patients were at rest, noninvasive monitoring of CO showed great variability between the non-invasive methods and frequently showed unacceptable errors in relation to procedures considered as “gold standard”, such as thermodilution. In an uncontrolled exercise scenario, both for the diagnosis of diseases and for the improvement of athlete conditioning, the non-invasive methods for CO monitoring seem to be more of a myth than a reality at present, when compared to the standard methods for calculating the CO.

Further studies are needed to determine CO through noninvasive methods at rest and during exercise. Our hope is that in the near future, and with the progress in technological development, the non-invasive monitoring of CO can be used in controlled and uncontrolled environments, in addition to the current perioperative scenario.
